# The complete chloroplast genome sequence of *Sargentodoxa cuneata*: genome structure and genomic resources

**DOI:** 10.1080/23802359.2020.1863870

**Published:** 2021-01-27

**Authors:** Xuejun Cui, Xingong Wang, Yanjie Wang, Qingjun Yuan, Ye Shen, Linde Liu

**Affiliations:** a School of Life Sciences, Ludong University, Yantai, China; b Department of Traditional Chinese medicine, Shandong College of Traditional Chinese Medicine, Yantai, China; c State Key Laboratory Breeding Base of Dao-di Herbs, National Resource Center for Chinese Materia Medica, China Academy of Chinese Medical Sciences, Beijing, China

**Keywords:** Chloroplast genome, *Sargentodoxa cuneata*, molecular markers, SSR

## Abstract

*Sargentodoxa cuneata* is used as traditional Chinese medicine. In this study, we report its complete chloroplast genome by Illumina pair-end sequencing. The total chloroplast (cp) genome size was 158,094 bp in length, containing a pair of inverted repeats of 26,132 bp, separated by large single-copy and small single-copy regions of 86,508 bp and 19,322 bp, respectively. The chloroplast genome of *S. cuneata* encodes 113 different genes, including 79 protein-coding genes, 30 transfer RNAs, and 4 ribosomal RNAs. A total of 84 perfect chloroplast microsatellites were analyzed in the *S. cuneata*. The majority of the SSRs in this chloroplast genome are mononucleotides (66.67%). The reconstructed phylogeny revealed that *S. cuneata* was sister to the remaining Lardizabalaceae.

*Sargentodoxa*, a monotypic genus of the Lardizabalaceae has often been placed in its own family, Sargentodoxaceae (Hoot et al. 1995). It consists of the single species, *Sargentodoxa cuneata* (Oliver) Rehder and E. H. Wilson, mostly confined to subtropical China (Wang et al. 2009). *S. cuneata* is used as traditional Chinese medicine or ethnic medicine (called Hongteng or Xueteng). Wild resources of this species have been seriously deteriorated due to years of over-harvesting, indicating an urgent need for reasonable conservation strategies. It is necessary to develop genomic resources for *S. cuneata* to provide intragenic information for its utilization and conservation. Because of the small and relatively constant size, conserved genome structure, and uniparental inheritance, chloroplast genomes provide a valuable genetic resource for phylogenetic analysis and species conservation (Dong et al. 2017; Dong et al. 2018). In this study, we sequenced and analyzed the chloroplast genome of *S. cuneata* based on the next-generation sequencing method (Li et al. 2018). The objective of this study was to gain a comprehensive understanding of the chloroplast genome of *S. cuneata* by describing its genome structure and features.

Sample of *S. cuneata* was collected from Xinyang, Henan province of China (31°49′24.2′′N, 114°06′50.6′′E). The voucher specimen (Specimen accession number: HNRC010701) was deposited at the herbarium of the National Resource Center for Chinese Materia Medica, China Academy of Chinese Medical Sciences. Total genomic DNA was extracted using mCTAB protocol (Li et al. 2013). The DNA from silica dried tissue was fragmented to construct 350 bp insert library following the manufacturer’s manual (Illumina Inc., San Diego, CA, USA). Approximately 4 Gb of raw data were generated with paired-end 150 bp read length. The chloroplast genome was assembled with GetOrganelle (Jin et al. 2020). Plastomes were annotated with Plann (Huang and Cronk 2015). The annotated genomic sequence had been submitted to GenBank with the accession number MT898426.

The size of the chloroplast genome of *S. cuneata* is 158,094 bp. The cp genome exhibits a quadripartite structure, which includes a pair of inverted repeats (IRa and IRb: 26,132 bp), and separated large single-copy (86,508 bp) and small single-copy (19,322 bp) regions. The GC content of the chloroplast DNA is 38.2%. The chloroplast genome of *S. cuneata* encodes 113 different genes, including 79 protein-coding genes, 30 transfer RNAs (tRNA), and 4 ribosomal RNAs (rRNA). Fifteen distinct genes contain a single intron while *ycf3* and *clpP* each contain two. Simple sequence repeats in the *S. cuneata* chloroplast genomes were detected using GMAT (Wang and Wang 2016) with the minimum repeats of mono-, di-, tri-, tetra-, penta- and hexa-nucleotides being set to 10, 5, 4, 3, 3, and 3, respectively. A total of 84 perfect cp microsatellites were analyzed in the *S. cuneata* . The majority of the SSRs in this chloroplast genome were mononucleotides (66.67%) and almost all of the mononucleotides (88.09%) are composed of A/T.

To estimate phylogenetic relationships of *S. cuneata* with other Lardizabalaceae species. Phylogenetic analysis was performed using the whole chloroplast genome sequence. The chloroplast genome sequences were aligned using MAFFT v7 (Katoh and Standley 2013). Ambiguous alignment regions were trimmed by Gblocks 0.91 b (Castresana 2002). The maximum-likelihood (ML) analyses were performed in RAxML v.8.1.24 (Stamatakis 2014). The statistical support for the branches was calculated by rapid bootstrap analyses with 1000 replicates. The reconstructed phylogeny revealed that *S. cuneata* was sister to the remaining Lardizabalaceae (Figure 1). The whole chloroplast genome sequences provided sufficient genetic information for species identification, phylogeny analysis, and conservation genetics.

**Figure 1.**
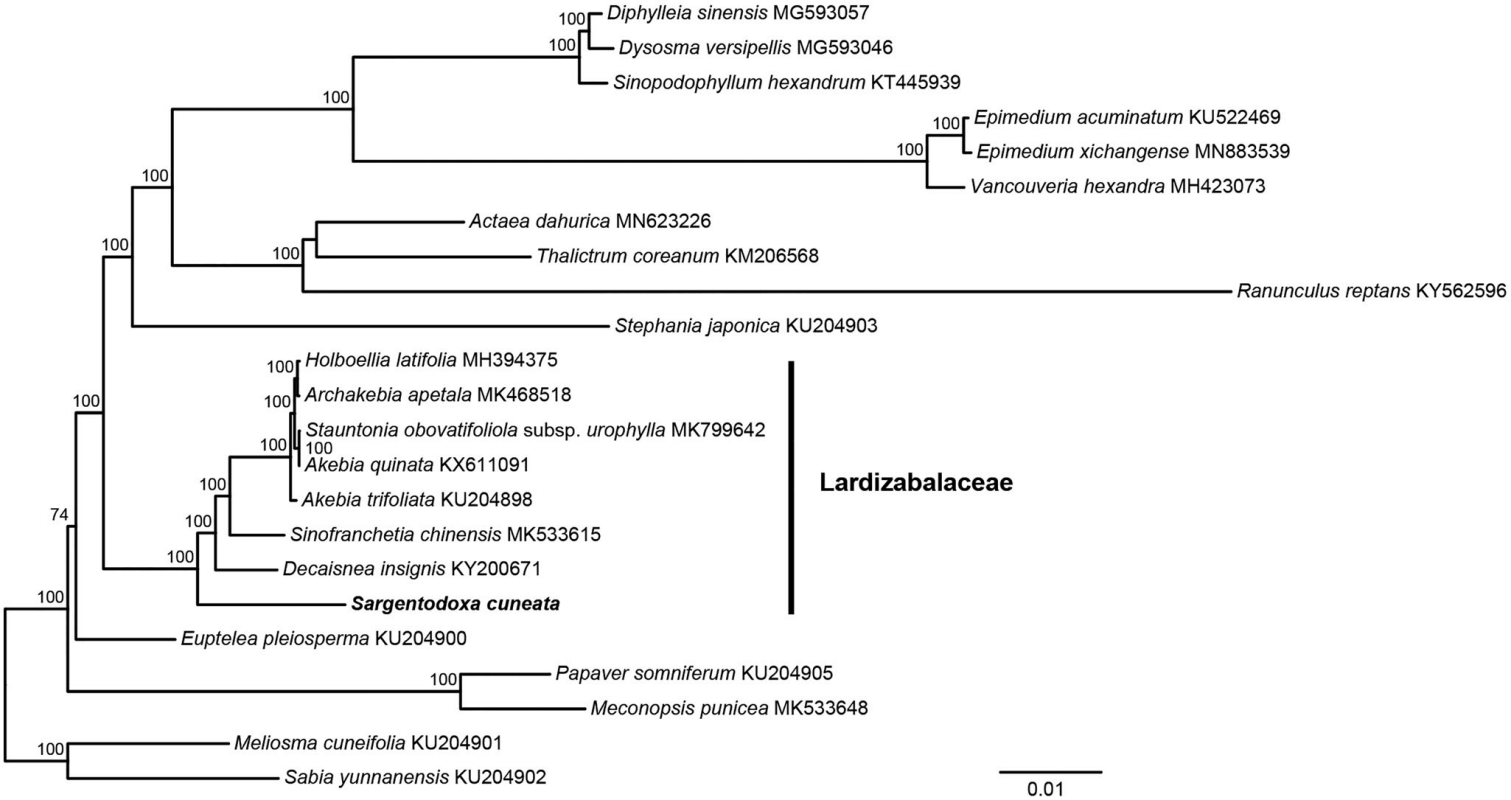
Maximum-likelihood tree of Ranunculales based on the complete chloroplast genome sequences. Bootstrap support values >50% are given at the nodes.

## Data Availability

The chloroplast genome sequence of the *S. cuneata* was submitted to GenBank of NCBI (https://www.ncbi.nlm.nih.gov). The accession number from GenBank is MT898426. The raw data have been deposited in SRA under accession no. PRJNA662211 (https://www.ncbi.nlm.nih.gov/sra/PRJNA662211)
